# Some Observations on Brain Anatomy and Brain Tumors

**Published:** 1898-10

**Authors:** 


### SOME OBSERVATIONS ON BRAIN ANATOMY AND BRAIN TUMORS.

ABSTRACT.

Dr. William C. Krauss, of Buffalo, read a paper at the ninety-second annual meeting of the Medical Society of the State of New York, Albany, Jan. 25, 1898, with the above title.

He called attention (1) to the difficulty in remembering the gross anatomy of the brain, and (2) to the almost universal presence of optic neuritis in cases of brain tumor.

He attempted to overcome the difficulty in regard to the anatomy of the brain by formulating the following rules, which are somewhat unique and original, and at the same time easily remembered.

RULE OF TWO. 1. The nerve centers are divided into two great divisions, (1) encephalon, (2) myelon. 2. The encephalon is divided into two subdivisions, (1) cerebrum, (2) cerebellum. 3. The cerebrum, cerebellum and myelon are divided into two hemispheres each, (1) right, (2) left. 4. The encephalon is indented by two great fissures, (1) longitudinal, (2) transverse. 5. Into these two great fissures there dip two folds of the dura, (1) falx cerebri, (2) tentorium cerebelli. 6. There are two varieties of brain matter, (1) white, (2) gray.

RULE OF THREE. 1. There are three layers of membranes surrounding the brain, (1) dura, (2) arachnoid, (3) pia. 2. Each hemisphere is indented by three major fissures, (1) sylvian, (2) rolandic or central, (3) parieto-occipital. 3. Three lobes, frontal, temporal and occipital, on their convex surface are divided into three convolutions each, superior, middle and inferior, or first, second, and third. 4. There are three pairs of basal ganglia, (1) striata, (2) thalami, (3) quadrigemina. 5. The hemispheres of the brain are connected by three commissures, (1) anterior, (2) medi, (3) post-commissure. 6. The cerebellum consists of three portions, (1) right, (2) left hemisphere, (3) vermes. 7. There are three pairs of cerebellar peduncles, (1) superior, (2) middle, (3) inferior. 8. The number of pairs of cranial nerves, in the classifications of Willis and Sommering, can be determined by adding 3 to the number of letters in each name, that of Willis making 9, and that of Sommering making 12 (or the name containing the more letters has the larger number of pairs of nerves, and vice versa). 9. The cortex of the cerebellum is divided into three layers of cells, (1) granular, (2) Purkinje's cells, (3) a molecular layer.

RULE OF FIVE. 1. Each hemisphere is divided externally into five lobes, of which four are visible, (1) frontal, (2) parietal, (3) temporal, (4) occipital; and one invisible, (5) insula (Isle of Reil). Roughly speaking, the visible lobes correspond to the bones of the cranium; that is, the frontal lobe is underneath the frontal bone, the parietal lobe beneath the parietal bone, etc. 2. The brain contains five ventricles, of which four are visible, the right and left, or first and second, the third and the fouth; and one invisible, the fifth, or pseudo-ventricle. 3. The cortex of the brain contains five distinct layers of ganglion cells.

Studying carefully a hundred casesof brain tumor in which an ophthalmoscopic examination had been made for the presence or absence of choked disc (optic neuritis), Dr. Krauss announced the following conclusions:

1. Optic neuritis is present in about 90 per cent of all cases of brain tumor.

2. It is more often present in cerebral than in cerebellar cases.

3. The location of the tumor exerts little influence over the appearance of the papillitis.

4. The size and nature of the tumor exerts but little influence over the production of the papillitis.

5. Tumors of slow growth are less inclined to be accompanied with optic neuritis than those of rapid growth.

6. It is probable that unilateral choked disc is indicative of disease in the hemisphere corresponding to the eye involved.

7. It is doubtful whether increased intracranial pressure is solely and alone responsible for the production of an optic neuritis in cases of brain tumor.—*The Philadelphia Medical Journal.*

---

"SOUTHERN MEDICAL RECORD."—Articles on "Points in Surgery and Medicine" have a character somewhat peculiar to this monthly. The editor seems to have a way of his own in managing his matter, and giving his magazine a special character.—*Phrenological Journal.*

